# Antibacterial composite coatings of MgB_2_ powders embedded in PVP matrix

**DOI:** 10.1038/s41598-021-88885-2

**Published:** 2021-05-05

**Authors:** P. Badica, N. D. Batalu, M. Burdusel, M. A. Grigoroscuta, G. Aldica, M. Enculescu, G. Gradisteanu Pircalabioru, M. Popa, L. G. Marutescu, B. G. Dumitriu, L. Olariu, A. Bicu, B. Purcareanu, L. Operti, V. Bonino, A. Agostino, M. Truccato, M. C. Chifiriuc

**Affiliations:** 1 National Institute of Materials Physics, Street Atomistilor 405A, 077125 Magurele, Romania; 2 University Politehnica of Bucharest, Splaiul Independentei 313, 060042 Bucharest, Romania; 3 Faculty of Biology and The Research Institute of the University of Bucharest (ICUB), University of Bucharest, Splaiul Independentei 91-95, Bucharest, Romania; 4 Biotehnos SA, Strada Gorunului, Nr. 3-5, Otopeni, Județul Ilfov, Romania; 5 Physics and Chemistry Departments, University of Turin, Via P. Giuria 1-7, 10125 Turin, Italy; 6 Academy of Romanian Scientists, 54 Splaiul Independentei, 050094 Bucharest, Romania

**Keywords:** Biomaterials - cells, Drug delivery

## Abstract

Three commercial powders of MgB_2_ were tested in vitro by MTS and LDH cytotoxicity tests on the HS27 dermal cell line. Depending on powders, the toxicity concentrations were established in the range of 8.3–33.2 µg/ml. The powder with the lowest toxicity limit was embedded into polyvinylpyrrolidone (PVP), a biocompatible and biodegradable polymer, for two different concentrations. The self-replenishing MgB_2_-PVP composite materials were coated on substrate materials (plastic foil of the reservoir and silicon tubes) composing a commercial urinary catheter. The influence of the PVP-reference and MgB_2_-PVP novel coatings on the bacterial growth of *Staphylococcus aureus* ATCC 25923, *Enterococcus faecium* DMS 13590, *Escherichia coli* ATCC 25922, *Pseudomonas aeruginosa* ATCC 27853, in planktonic and biofilm state was assessed in vitro at 6, 24, and 48 h of incubation time. The MgB_2_-PVP coatings are efficient both against planktonic microbes and microbial biofilms. Results open promising applications for the use of MgB_2_ in the design of anti-infective strategies for different biomedical devices and systems.

## Introduction

The opportunistic and nosocomial agents, such as ESCAPE (*Enterococcus faecium, Staphylococcus aureus, Clostridium difficile, Acinetobacter baumannii, Pseudomonas aeruginosa,* and Enterobacteriaceae) pathogens and *Candida albicans* represent one of the most important global threats for the public health. They have the ability to adhere and develop biofilms on live tissues and implanted medical devices, consequently producing biofilm associated infections^1^. Biofilms exhibit a particular form of resistance, the phenotypic resistance or tolerance. They also have recalcitrance, i.e. the ability to survive in the presence of high concentrations of antibiotics^2^.

The multiple negative consequences of biofilms development in the clinical sector underlines the need to recognize the problems contributing to poor outcomes and high costs. It also highlights the necessity of a multidisciplinary effort to prevent, combat, or eradicate biofilms. The antimicrobial strategies can be divided in microbiostatic/microbicidal based agents or on antipathogenic agents. The first ones involve the use of agents which inhibit or kill microorganisms, while the antipathogenic ones target the expression of virulence factors (e.g., adherence capacity, toxigenicity) and of their regulators (Quorum Sensing inhibitors)^3^. The antibiofilm strategies belong to both categories of the antimicrobial ones.

Nanotechnology and nanomaterials are of much interest for the development of new antimicrobial approaches, based on either novel biomaterials or on improving the biological properties of the existing ones. Currently, in a sustainable and eco-friendly driven approach, many studies are directed to design both clinically and environmentally safe nanomaterials (NMs) for antimicrobial applications. The NMs act as antimicrobial and antibiofilm agents. They can have additive or synergetic effects in combinations with antibiotics or other antimicrobials^4–7^. NMs are also useful as drug delivery for targeted release to the site of infection and as components of composites including stimuli-responsive coatings, or modified hybrid materials^8–10^. Many physico-chemical properties, such as the type of the nanomaterial, size, morphology, specific surface-area-to-volume ratio, surface charge, concentration, behavior in biological medium and pH, stability and others are conditioning their antibiofilm effect. All these factors influence the contact with the biofilm matrix and biofilm embedded cells, affecting the release of reactive oxygen species, of antimicrobial ions or of the loaded bioactive compounds^11^. NMs can be modified through functionalization to increase their efficacy and biocompatibility^12^. They can simultaneously attack multiple microbial targets, thereby the risk of emergence of resistance is low. The NMs can interfere with different stages of biofilm development, i.e., with single-cell adherence, multiplication, and colonization of the substrate, with biofilm maturation, and with biofilm dispersion. They can interact with the planktonic cells, inhibiting either the initial adhesion to a substrate and the dispersion, or with the biofilm matrix by facilitating the biofilm penetration, drug release, or further interaction with the biofilm cells^13^.

Metals (silver, copper, gold, chromium), metal oxides (Al_2_O_3_, CeO_2_, Co_3_O_4_, Cr_2_O_3_, CuO, In_2_O_3_, Fe_2_O_3_, MgO, Mn_2_O_3_, NiO, Ni_2_O_3_, SiO_2_, TiO_2_, ZnO, ZrO_2_, Y_2_O_3_, etc.), metal hydroxides, such as Mg(OH)_2_, and metal halides nanoparticles (NPs) are among the most studied NMs for antimicrobial effects, due to their intrinsic antimicrobial features^11,14–18^. They exhibit both microbicidal and microbiostatic effects caused by membrane lesions due to the direct contact with NPs and the release of free metal ions, the proteins inactivation, the nucleic acids damage, the release of reactive oxygen species (ROS), and stimulation of the host immune system.

The delivery of active NPs inside biofilms is possible by using different delivery systems, including polymers. Polyvinylpyrrolidone (PVP), also called polyvidone or povidone, is a biocompatible, non-toxic, biodegradable, hydrophilic polymer with good binding properties and with a stabilizing effect on suspensions and emulsions. PVP is recognized as safe by the Food and Drug Administration (FDA). Considering also that it has other unique physical and chemical features, e.g. it is chemically inert, but soluble in water and alcohol, can have different morphologies, it is colorless, temperature-resistant and pH-stable, PVP is largely used for biomedical applications^19–21^.

In this paper, we continue our previous studies on antimicrobial activity of MgB_2_ powders^22,23^ and we investigate their potential when embedded in PVP-based polymeric coatings for fabrication of improved plastic medical devices, more resistant to microbial colonization and thus, less probable to induce biofilm-associated infections. As substrate materials we use a flexible plastic foil of a urinary catheter reservoir and silicon tubes from the same commercial device. The challenges in using catheter devices and criteria for their improvement are reviewed in Refs.^24,25^. The MgB_2_ compound is degradable in water^26,27^ and it results that the composite coatings of PVP- MgB_2_ are biodegradable and self-replenishing. Other different factors that recommend MgB_2_ for biomedical application are: (i) as already mentioned, Mg is a an antimicrobial material, but also boron in the form of boric acid or sodium salts of boron (borax, disodium tetraborate) is an effective antiseptic, bactericidal, insecticidal, herbicidal and cleaning agent used in detergents (^28^ and therein Refs.); (ii) on the other hand, both Mg and boron are involved in the metabolism of humans from bone growth to wound healing and their amount in the body is relatively high; boron has positive effects and it is used in treatment of cancer and infections; (iii) MgB_2_ is a light weight compound with bulk density of 2.63 g/cm^3^ that is close to that of the bones; (iv) the mechanical properties of MgB_2_ are similar to those of ceramic materials and of the bones; (v) MgB_2_ has metallic conduction and when decomposes it releases positive (Mg) and negative (B) ions impacting local pH and interaction with negatively charged cellular wall; (v) there is a family of MgB_y_ (MgB_4_, MgB_7_, MgB_12_, MgB_19_) phases that may prove of interest to control the bioprocesses depending on application requirements.

## Experimental

### Physico-chemical characteristics of MgB_2_ raw powders

Commercial raw powders of MgB_2_ were produced by LTS Research Laboratories Inc (LTS), Alfa Aesar (AA), and CERAC Inc (CER). The powders have very different physico-chemical properties. Although results will be presented elsewhere^23^, according to X-ray diffraction measurements and Rietveld analysis, we mention that powders were composed of the main phase MgB_2_ and of the secondary phases MgO and MgB_4_. The highest MgB_2_ amount was measured in LTS (97 wt. % MgB_2_) followed by Alfa Aesar (88 wt. % MgB_2_), and Cerac (80.3 wt. % MgB_2_). In the same order, the amount of residual unreacted metallic Mg (1.2–0 wt. %) decreases, while the amount of secondary phases MgO (1.8–7.9 wt. %) and MgB_4_ (0–11.8 wt. %) increases. The crystallite size of the powders is comparable (105–113 nm). In the LTS and Alfa Aesar powders, the amount of carbon (denoted *y*) substituting for boron in the crystal lattice of MgB_2_ is not much different (*y* = 0.0011/0.0015). In the Cerac powder *y* is more than double (0.0039), considering the chemical formula Mg(B_1-*y*_C_*y*_)_2_. The powders have a bimodal particle size distribution. The LTS powders, followed by Alfa Aesar and Cerac, show high and low intensities of the peaks for the small and large size fractions, respectively. The large size fraction is composed of stable particle agglomerates. A higher fraction of small particles explains the lower flowability and a higher rate of pH increase (for powders immersed in water) towards saturation of LTS followed by Alfa Aesar and Cerac. The pH saturation is within a narrow range of 9.9 and 10.1. The surface of the particles was studied by TEM and it was appreciated that surfaces are relatively clean, lacking oxides and impurities.

### MgB_2_ raw powders cytocompatibility tests

The cytotoxic (loss of viable cells) effect of MgB_2_ powders on fibroblast HS-27 (skin) cell line is assessed by observing the correlated change in cell viability (MTS test^29^) and enzymatic activity in the culture medium (LDH test). Since the main function of fibroblasts is to maintain the structural integrity of connective tissue by synthesizing extracellular matrix components (particularly, type I collagen), the HS-27 line is a representative standard dermal cell line for testing primarily the potential of MgB_2_ for topical use and related applications. In this work the quantitative results of this test are used as input guiding data for the design of the MgB_2_-PVP coatings compositions.

Cells were incubated with MTS (Kit: CellTiter 96 AQueous One Solution Cell Proliferation Assay, Promega) colorimetric assay (3-(4,5-dimethylthiazol-2-yl)-2,5-diphenyltetrazolium bromide)^30,31^. The compound is reduced by living cells to purple and water-soluble formazan. The conversion of MTS to formazan takes place under the action of enzymes (dehydrogenases) from metabolically active cells. The absorbance of formazan at 490 nm is measured spectrophotometrically (TriStar Berthold Technologies) directly in the 96-well plates. The amount of produced formazan is quantified by absorbance which is directly proportional to the number of living cells in the culture.

Lactate dehydrogenase (LDH) is a cytosolic enzyme from the cytoplasm of any cell. Damage to the cell membrane causes the leak of LDH into the extracellular fluid. In vitro release of LDH provides an accurate way to measure cell membrane integrity and, implicitly, cell viability^32^. To test the cytotoxic effect of MgB_2_ on HS-27 cell culture, cells are exposed to increasing concentrations of MgB_2_ solutions. The MgB_2_ solutions used in the MTS and LDH experiments are the same and their preparation is addressed in the next paragraphs. The release of LDH in the cell culture supernatant correlates with the cytotoxicity^33,34^ and it is measured by a test in which two coupled enzymatic reactions, catalyzed by LDH and diaphorase, take place. The reactions convert a tetrazolium salt into a red formazan compound. Absorbance at 490 nm is measured spectrophotometrically (TriStar Berthold Technologies). The kit used in our experiments was CytoTox 96 Non-Radioactive Cytotoxicity Assay (Promega).

For each powder (LTS, Alpha Aesar and Cerac) two stock solutions (8.3 mg/ml and 33.3 mg/ml) in ethanol (99.8%) were prepared. Decimal dilutions were further used in the test. After 34 h adhesion time in 96-well culture plates, fibroblasts were treated with MgB_2_ solutions for 48 h. The protocol involving an incubator for cell cultures with a humid atmosphere and 5% CO_2_ at 37 °C) was applied, where the culture medium was composed of DMEM, 1% antibiotic/antifungal, 10% fetal bovine serum. The absorbance of the samples was related to that of the environmental control, the solvent control, and the cell control.

In the MTS and LDH tests, three experiments were performed using samples in triplicate. The absorbance ratio *R* of solutions with different concentrations containing treated and untreated cells in the MTS (*R*_MTS_) and LDH (*R*_LDH_) tests was plotted vs. concentration of MgB_2_ solutions. For an increasing concentration, a decrease of *R*_MTS_ (equivalent to less formazan produced by fewer living cells) and an increase of *R*_LDH_ (equivalent to more LDH as a result of a lower metabolic activity due to more damaged cells) determine the cytotoxicity threshold.

### Fabrication of composite MgB_2_-PVP coatings

**Figure 1:**
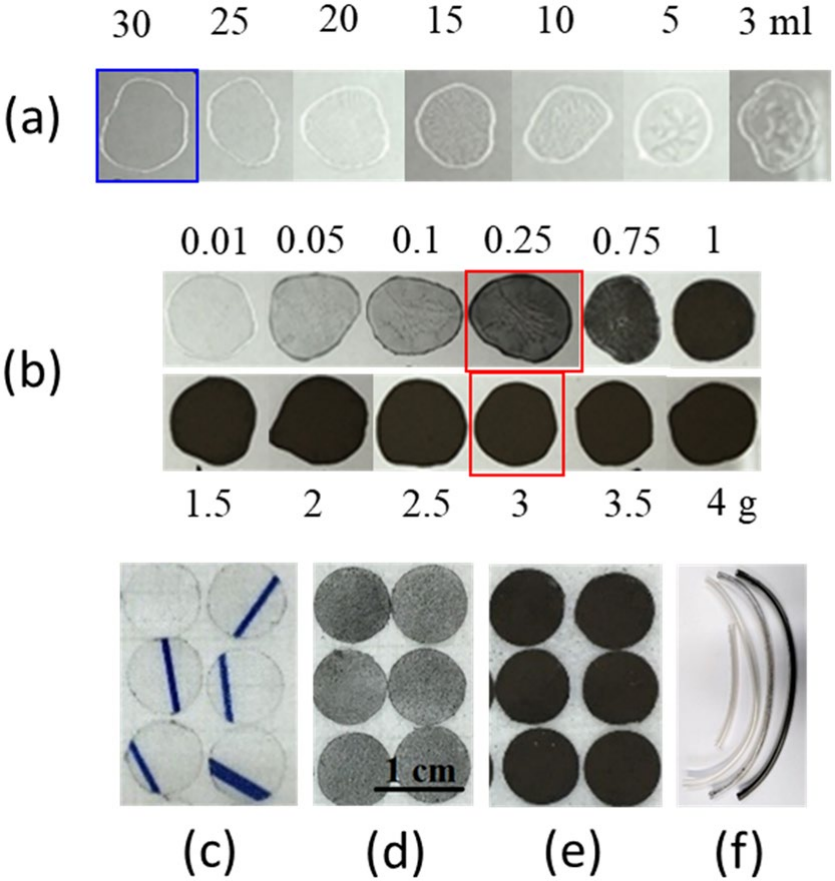
Coatings fabrication stages: (**a**) coatings of PVP with different concentrations (0.5 g of PVP in 3–30 ml of ethanol) on a glass substrate; (**b**) coatings of MgB_2_-PVP with different concentrations (0.01–4 g of MgB_2_ introduced into a solution of 0.5 g PVP/30 ml ethanol) on a glass substrate (see text); (**c**–**e**) coatings on the polymer foil of the catheter reservoir of PVP^foil^ (0.5 g PVP/30 ml ethanol), (MgB_2_-PVP)^foil^_0.25_ (0.25 g MgB_2_/0.5 g PVP/30 ml ethanol), (MgB_2_-PVP)^foil^_3_ (3 g MgB_2_/0.5 g PVP/30 ml ethanol), respectively; (**f**) (from left to right) pristine silicon tube of a catheter and coatings on the catheter silicon tube of PVP^tube^ (0.5 g PVP/30 ml ethanol), (MgB_2_-PVP)^tube^_0.25_ (0.25 g MgB_2_/0.5 g PVP/30 ml ethanol), (MgB_2_-PVP)^tube^_3_ (3 g MgB_2_/0.5 g PVP/30 ml ethanol).

The LTS powder with the highest antimicrobial activity^23^ was selected for fabrication of MgB_2_-PVP composite coatings. Commercial polyvinylpyrrolidone (PVP) powder (Sigma Aldrich, the monomer N-vinylpyrrolidone, with chemical formula C_6_H_9_NO and molar weight M_w_ = 1,300,000 g/mol) was dissolved in ethanol at room temperature. The amount of the PVP powder immersed in ethanol was constant, 0.5 g, and the volume of ethanol was modified from 3 to 30 ml. Droplets of each solution were placed on a glass substrate (Fig. 1a). A higher uniformity of the coating and less trapped gas particles were noticed for a lower concentration; hence the lowest tested concentration (0.5 g PVP/30 ml ethanol) was selected for further processing stages. The PVP polymer is also soluble in water and this property will be used to release the active MgB_2_ particles when in contact with bacteria.

**Figure 2:**
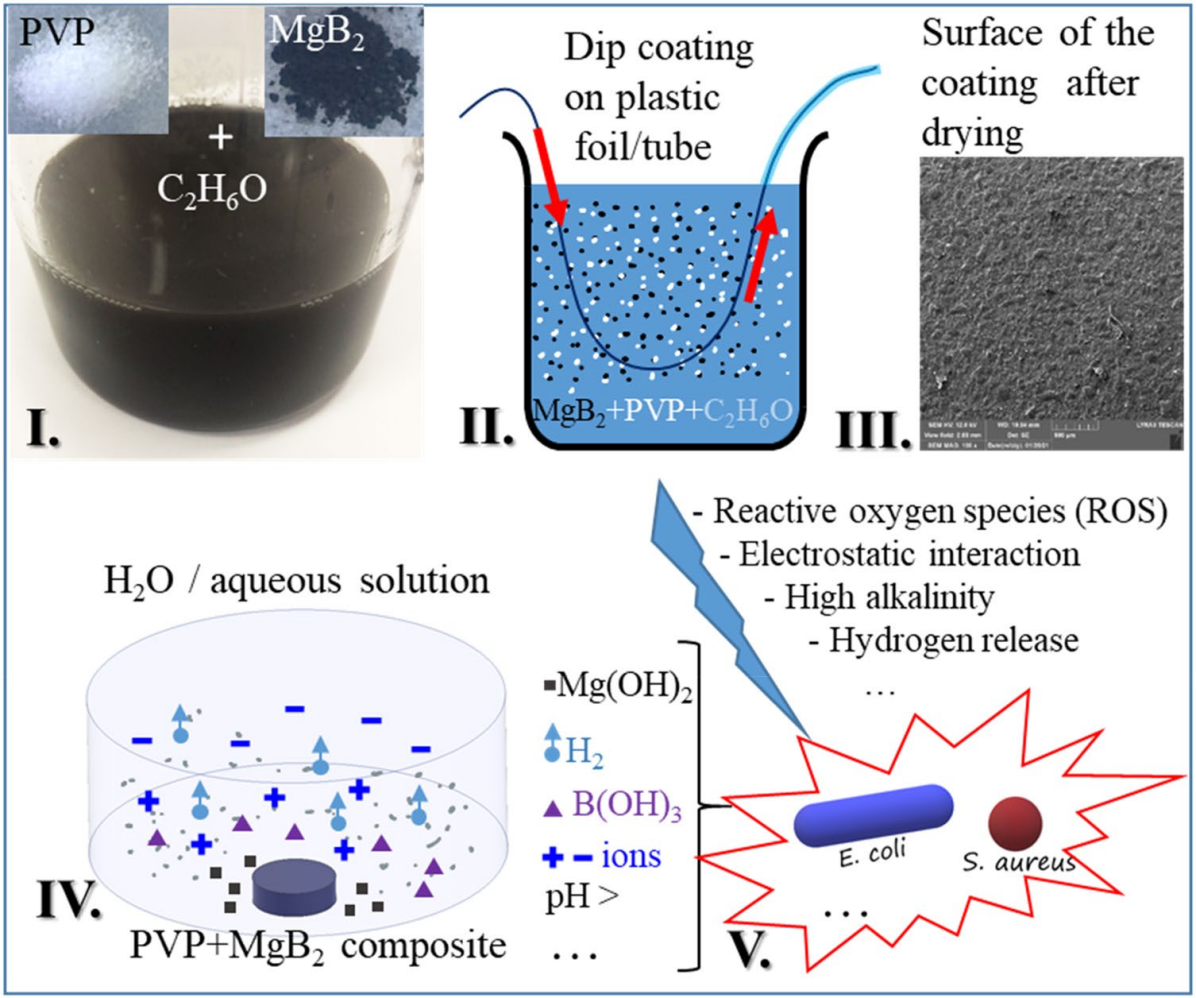
Preparation of composite MgB_2_-PVP coatings (**I**–**III**), release of active MgB_2_ from the composite coating and its decomposition in the presence of water/aqueous solution (**IV**), and the antimicrobial effect through possible mechanisms on different microbes. SEM image from III is taken on the sample (MgB_2_-PVP)^foil^_0.25_.

The MgB_2_ powder was introduced into as-prepared PVP-ethanol solution and mixed ultrasonically for few minutes, and a colloidal black solution was obtained (Fig. 2I). MgB_2_ is not soluble in alcohols^35^. The amount of the MgB_2_ powder varied from 0.01 to 4 g. Droplets of the colloidal solution were placed on a glass substrate and dried naturally under ambient conditions (Fig. 1b). The uniformity, bubbles size and amount, and adherence of the coatings were observed. Two concentrations, 0.25 and 3 g per initial solution (0.5 g PVP/30 ml ethanol) were selected. Considering the density of PVP of 1.2 g/cm^3^, the selected concentrations of MgB_2_ in PVP are 60 mg/ml and 720 mg/ml, respectively. For MgB_2_ concentrations in PVP greater than 720 mg/ml, coatings are not uniform, showing a high level of granularity that makes them easily breakable and removable from the substrate.

The plastic foil of a reservoir and the silicon tube, both from a commercial urinary catheter, were used as substrates for coatings fabrication (Fig. 1c–f). Coatings of bare PVP as reference samples were also deposited. The composite films on the plastic foils were obtained by dip coating and subsequent drying at room temperature (Fig. 2II,III). They were cut into samples of 8 mm in diameter (Fig. 1c–e). The PVP-MgB_2_ composite covered both sides of the foil substrate. Coloidal solutions of MgB_2_/PVP/ethanol were poured into a silicon tube and, after drying, a coating was obtained inside the tube (Fig. 1f). The inner/outer diameter of the tube was 4.5/6 mm. After coating, the tube was cut into pieces of 1 cm in length.

Coated samples were labeled as: PVP^foil^ and PVP^tube^ (0.5 g PVP/30 ml ethanol), (MgB_2_-PVP)^foil^_0.25_ and (MgB_2_-PVP)^tube^_0.25_ (0.25 g MgB_2_/0.5 g PVP/30 ml ethanol), (MgB_2_-PVP)^foil^_3_ and (MgB_2_-PVP)^tube^_3_ (3 g MgB_2_/0.5 g PVP/30 ml ethanol).

**Figure 3:**
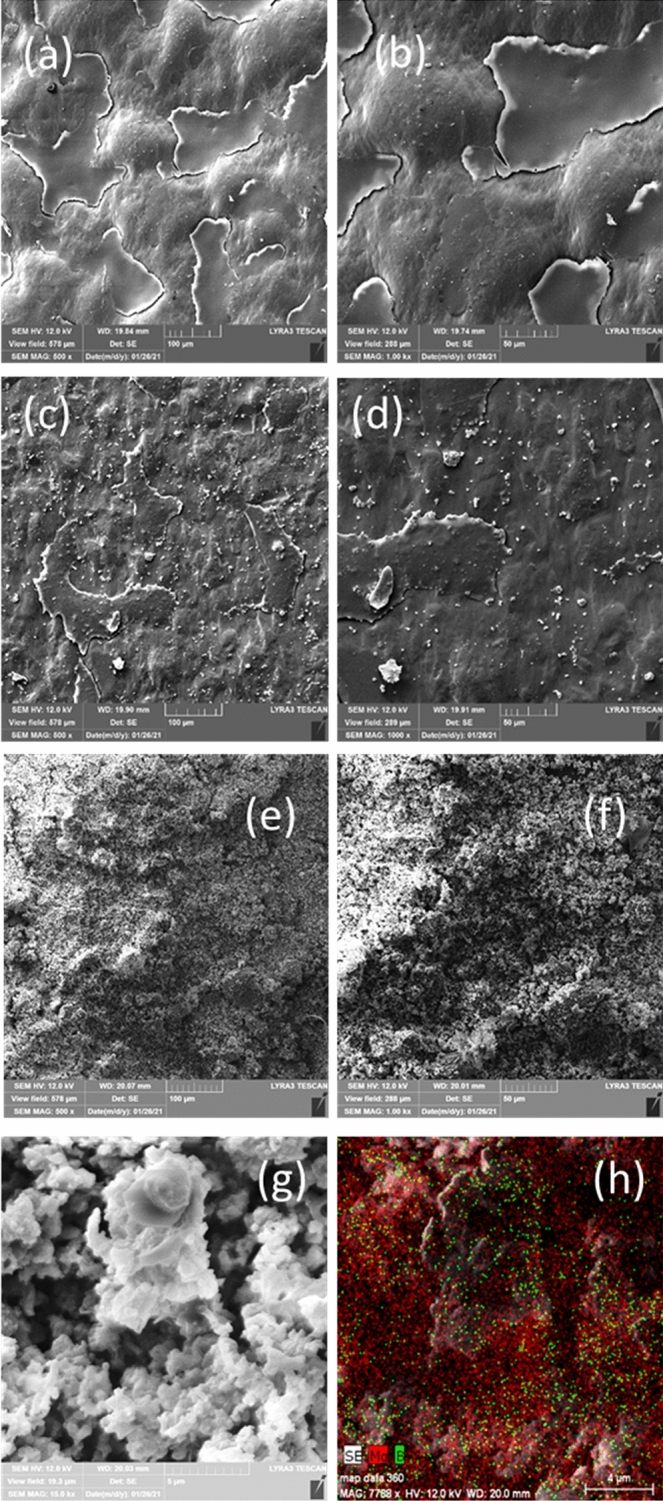
SEM images at two magnifications (× 500, × 1000) on MgB_2_-PVP films coated on plastic foil: (**a**,**b**) sample PVP^foil^; (**c**,**d**) sample (MgB_2_-PVP)^foil^_0.25_; (**e**,**f**) sample (MgB_2_-PVP)^foil^_3_. Secondary electron image from (**g**) presents a detail at high magnification (× 15,000) of sample (MgB_2_-PVP)^foil^_3_, while (**h**) is a red–green–blue image obtained by overlapping the elemental EDS maps of Mg and B measured on image from (**g**).

The microstructure by scanning electron microscopy (SEM, Lyra 3XMU/Tescan) of the composite MgB_2_-PVP coatings (covered with Au) on the plastic foil can be visualized in Fig. 3. The surface morphology of the composite film changes with addition of a higher amount of MgB_2_ powder in the coating. The presence of MgB_2_ influences drying of the coating and its roughness. While drying improves and roughness decreases for sample (MgB_2_-PVP)^foil^_0.25_ (Fig. 3c,d) when compared to PVP^foil^ (Fig. 3a,b), addition of a higher amount of MgB_2_ as for sample (MgB_2_-PVP)^foil^_3_ (Fig. 3e,f) produces a very rough and granular surface. In the sample (MgB_2_-PVP)^foil^_3_ one can actually clearly distinguish grains with plate like or irregular morphology (Fig. 3e,f) that are gathered into agglomerates. The morphology of the particles in the composite film is similar to that observed in the raw LTS MgB_2_ powder^23^. Elemental mapping shows the presence of B and Mg, distributed uniformly at the scale of our observation (Fig. 3g,h). This result and short-time sonication for low energy (200 W) suggests that the integrity of MgB_2_ was preserved during its processing in the PVP-ethanol solution for preparation of the composite film. However, it is noteworthy that ultrasonication for 30 min at room temperature of MgB_2_ in water produced through exfoliation Mg-deficient hydroxyl-functionalized nanosheets of MgB_2_^36^, while processing of MgB_2_ in acetonitrile mixed with an ion-exchange resin leads to formation of borophane sheets (hydrogenated borophene, HB)^37^.

### In vitro assay of antibacterial activity of MgB_2_-PVP coatings

The antimicrobial activity of the composite MgB_2_-PVP coatings (on foil and tubes) was tested against bacteria in planktonic and biofilms growth states (*Staphylococcus aureus* ATCC 25923, *Enterococcus faecium* DMS 13590, *Escherichia coli* ATCC 25922, *Pseudomonas aeruginosa* ATCC 27853).

In the case of planktonic bacteria, microbial suspensions of McFarland 0.5 (corresponding to 1.5 × 10^8^ CFU/ml) were prepared from 24 h cultures in sterile saline solution. The obtained suspensions were further diluted 1:100 in broth. Each composite MgB_2_-PVP sample was introduced into a well of a 24-well plate. The plate was inoculated with the prepared microbial suspension (750 µl). The final inoculum size was of 5 × 10^5^ CFU/ml for each tested coating. Untreated microbial cultures and sterile broth wells served as positive and negative controls. The inoculated 24 well-plates were incubated at 37 °C, in aerobic conditions for 6 h, 24 h, and 48 h. After incubation for the respective time intervals, the density of the microbial broth culture was determined by plating serial ten-fold dilutions prepared in sterile saline onto PCA (Plate Count Agar) and counting the number of colonies developed at different dilutions. Results were further used to determine the number of colonies forming units (CFU/ml).

In the case of biofilms, coatings were placed in contact with an inoculum of 5 × 10^5^ CFU/ml obtained from each microbial strain and incubated for 6, 24, and 48 h. After incubation, each material was carefully washed to remove the non-adherent bacteria and sonicated in sterile saline solution to disperse the biofilm, and serial decimal dilutions were seeded on agar plates to determine the CFU/ml as presented above.

All assays were done in triplicate.

## Results

### Cytocompatibility of MgB_2_ powders

**Figure 4:**
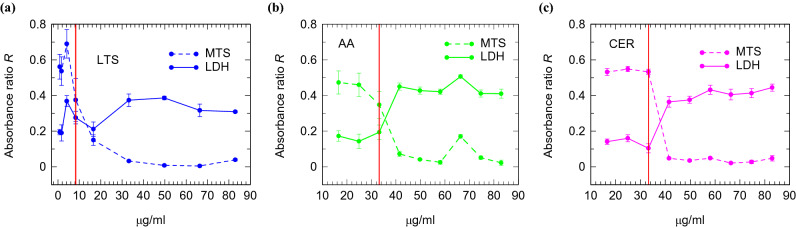
The absorbance ratio *R* in MTS and LDH cytotoxicity tests on HS27 cell line for dilutions prepared from stock solutions of 8.3 mg/ml of three types of MgB_2_ powders.

All dilutions (66–333 µg/ml) of stock solution with concentration 33.3 mg/ml and for the three investigated MgB_2_ powders (LTS, Alfa Aesar and Cerac) have shown cytotoxicity on the H27 cell line. The effect of dilutions with smaller concentrations (0.83–83 µg/ml) prepared from the stock solution 8.3 mg/ml are presented in Fig. 4. Concentrations of MgB_2_ solutions below 8.3, 33.2, and 33.2 µg/ml for the powders LTS, Alfa Aesar, and Cerac are compatible with the metabolism of fibroblasts without a toxic impact. In other experiments with different cells^38^ the toxicity limit was in the range of 50–100 µg/ml. The LTS powder has a lower biocompatibility than Alfa Aesar and Cerac powders.

Higher cytotoxicity of LTS comparative to the other MgB_2_ investigated powders can be inferred from its low tendency to form large agglomerates, clean particle surfaces, high amount of MgB_2_ phase, and high pH-increase rates^23^. Different activity of different MgB_2_ powders enables the possibility of a time and space-controlled reaction of MgB_2_ with biological medium, depending on the application requirements, so that the toxic effect is minimized, and antibacterial impact is maximized.

For design purposes of the MgB_2_-PVP coatings, apart from the as-evaluated cytotoxicity concentrations, we considered the minimum inhibitory concentrations for the growth in the presence of LTS, Alfa Aesar and Cerac MgB_2_ powders of different reference microbes (*Staphylococcus aureus* ATCC 25923, *Staphylococcus aureus* ATCC 6538, *Pseudomonas aeruginosa* ATCC 27853, *Escherichia coli* ATCC 25922, and *Candida albicans* ATCC 10231) in the planktonic (MIC) and biofilm (MICB) states reported in Ref.^23^. Namely, the MIC and MBIC values ranged between 0.31 and 1.25 mg/ml and between 0.039 and 0.62 mg/ml, respectively. The highest antimicrobial activity was found for LTS and the lowest for Cerac. The MgB_2_ powders were active also against planktonic cells and biofilms of 29 methicillin resistant clinical *S. aureus* isolates and 33 vancomycin resistant *E. faecium/faecalis* strains collected from clinical souces. The MIC values of 0.15–2.5 mg/ml were determined for different fungi collected from the heritage buildings and objects^38^.

### Antibacterial activity of MgB_2_-PVP composite coatings

**Figure 5:**
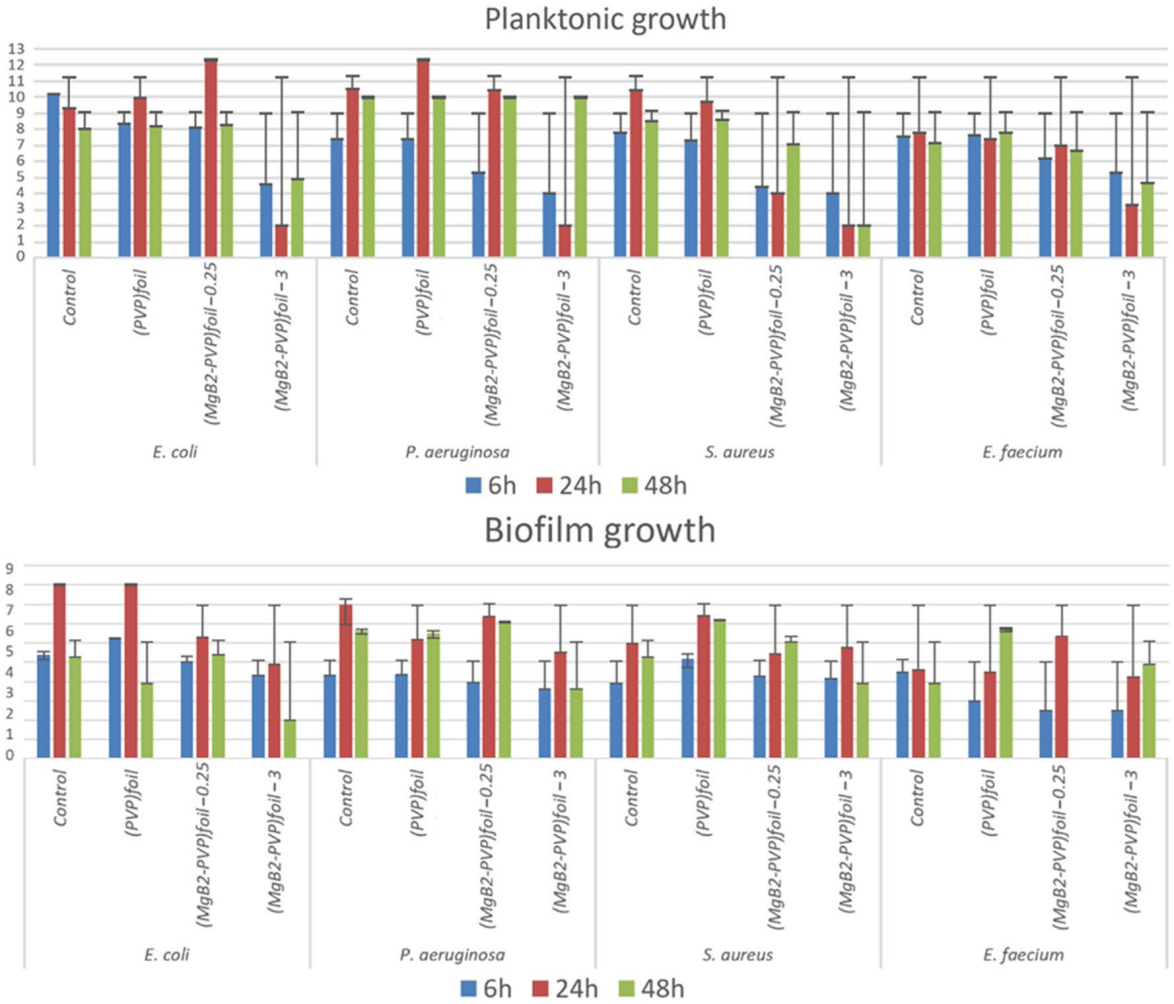
The number of viable microbial cells in log_10_(CFU/ml) for samples in the form of coatings on the plastic foil (i.e. samples PVP^foil^, (MgB_2_-PVP)^foil^_0.25_, (MgB_2_-PVP)^foil^_3_) tested with microbes in plaktonic and biofilm growth states.

**Figure 6:**
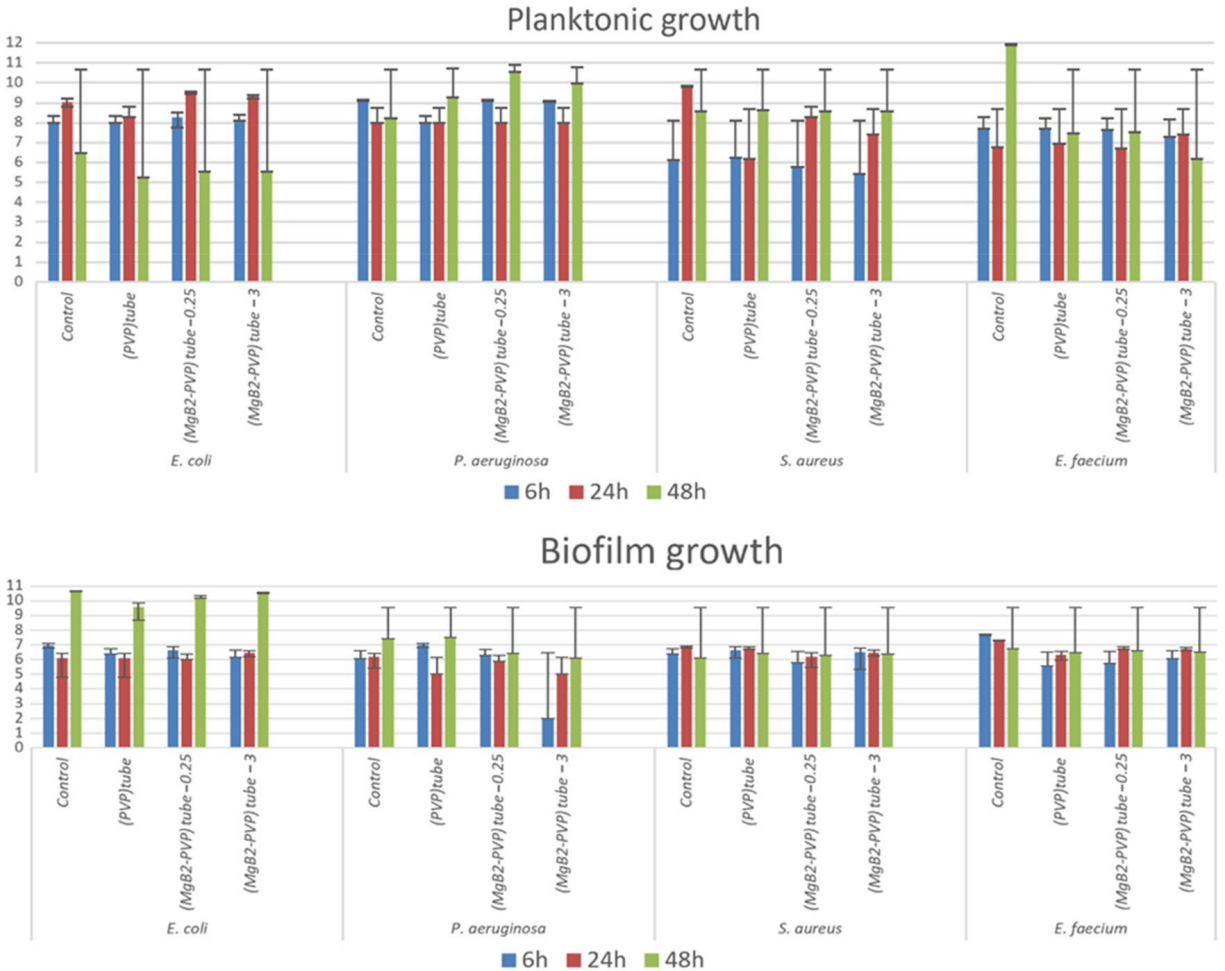
The number of viable of microbial cells in log_10_(CFU/ml) for samples in the form of coatings on the silicone tube (i.e. samples PVP^tube^, (MgB_2_-PVP)^tube^_0.25_, (MgB_2_-PVP)^tube^_3_) tested with microbes in planktonic and biofilm states.

The influence of PVP and MgB_2_-PVP coatings on the bacterial growth in planktonic state (in the liquid culture medium) and biofilm state (adhered on the surface of plastic foil or silicon catheter) was assessed at 6, 24, and 48 h of bacterial strains incubation with the tested samples. Results are presented in Figs. 5 and 6.

The general trend was that the MgB_2_-PVP samples exhibited an evident inhibitory activity on the planktonic growth of all tested strains, starting with 6 h of incubation, exacerbating after 24 h, and maintaining it at 48 h (Figs. 5, 6).

The inhibitory effect of the planktonic growth was more intense in the case of the samples (MgB_2_-PVP)^foil^_3_ containing the highest concentration of MgB_2_. In the case of the bacterial cells adhered on the surface of the functionalized plastic samples, the anti-biofilm effect was strain specific, but however, less evident than in the case of planktonic cells. The *E. coli* biofilm development was inhibited at all three time points by the (MgB_2_-PVP)^foil^_3_. The same sample inhibited the *P. aeruginosa* and *S. aureus* biofilm at 24 h and 48 h, respectively. In the case of *E. faecium,* all samples inhibited the initial cells adherence to the plastic foil, quantified after 6 h of incubation, and the (MgB_2_-PVP)^foil^_3_ exhibited also a slight inhibitory effect against the 24 h biofilm (Fig. 5). The comparative behaviour of planktonic and adhered cells developed in the presence of MgB_2_-functionalized plastic-foil samples suggests that the coatings exhibit their antimicrobial effect mostly by releasing the MgB_2_ in the active form.

The rate of the bacterial strains growth in the presence of the MgB_2_-functionalized silicon tubes was different from that recorded on the plastic samples, with no significant inhibition of the planktonic growth, except a slight decrease of the number of the viable cells of *S. aureus* after 24 h of incubation and of *E. coli* and *E. faecium* after 48 h of incubation. No significant differences between the PVP and MgB_2_-PVP samples (Fig. 6) were noted. In the case of the bacterial cells adhered on the surface of the MgB_2_-functionalized silicon tubes, the (MgB_2_-PVP)^tube^_3_ inhibited the *P. aeruginosa* biofilm development, with the anti-biofilm effects intensity decreasing in time from 6 to 48 h. The (MgB_2_-PVP)^tube^_0.25_ sample has shown a slight tendency for inhibition of the *S. aureus* biofilm development, the effect being noticable after 6 h of incubation. All samples slightly inhibited the development of *E. faecium* at all three incubation times. As for *S. aureus*, the effect is stronger at 6 h.

Results indicate that MgB_2_-containing samples on foils are more active than the coatings on silicon tubes. To understand it, further research is necessary. Among the possible reasons there could be how the shape of the sample influences the contact with the cell browth, the release of MgB_2_ and its interaction with the cells. A second aspect that could play a role is the fact that adherence of the coatings on the silicon tube is poor when compared to that on the foil. This is especially problematic for coatings with a high concentration of MgB_2_, i.e. for the samples (MgB_2_-PVP)^tube^_3_.

## Discussion

Considering the data presented in “Cytocompatibility of MgB_2_ powders” it results that selected MgB_2_ powder for coatings fabrication, LTS, has the lowest toxicity limit, corresponding to the highest cytotoxicity. At the same time, this powder is the most active one from the viewpoint of antimicrobial efficiency, i.e. the MIC and MBIC values are the lowest^23^. To understand the context of our antimicrobial assessment from “Antibacterial activity of MgB_2_-PVP composite coatings” and to provide some guiding lines in designing MgB_2_-PVP coatings for future different applications, we present some useful details in the following paragraphs.

The thickness of the coatings was estimated from optical measurements at ~ 200 µm. The volume of the coatings for the foil and tube samples was 0.02 and 0.028 ml, respectively. When using 0.25 g and 3 g of MgB_2_ to fabricate the samples, this results into a concentration of 0.6 and 7.2 mg/ml of the powder in the volume of PVP dissolved in ethanol (density of PVP was taken as 1.2 g/cm^3^ and density of MgB_2_ as 2.6 g/cm^3^). Rescaling for the volume of the coating on the foil, the (MgB_2_-PVP)^foil^_0.25_ and (MgB_2_-PVP)^foil^_3_ samples contain 9.75 and 38.2 mg of MgB_2_, respectively. A similar calculation for the (MgB_2_-PVP)^tube^_0.25_ and (MgB_2_-PVP)^tube^_3_ samples gives the values 13.65 and 53.5 mg, respectively.

To obtain a correct intepretation of our data, we should also take into consideration the PVP behavior in water. To this purpose, a bulk rectangular piece of PVP (0.5 cm × 0.5 cm × 0.1 cm) was prepared by casting a viscous solution of PVP in ethanol and subsequent drying in the air. The PVP sample with a volume 0.025 ml was introduced in water (50 ml). It dissolved in 3 h at room temperature, without steering. The dissolution rate of PVP in water is 0.0083 ml/h.

In the in vitro antimicrobial tests addressed in “Antibacterial activity of MgB_2_-PVP composite coatings”, samples were fully immersed in 750 µl of culture medium. The incubation time of 6, 24, and 48 h is higher than the dissolution time of PVP in water of 3 h. One also observes that the volume of the bulk PVP sample (0.025 ml) is comparable to that of the coatings (0.02 and 0.028 ml on the foil and on the tube, respectively). This suggests that the entire amount of MgB_2_ powder in the coating was available to interact with the bacterial cells from the broth. Hence, the concentration of MgB_2_ in the cell broth was as follows: 13, 50.9, 18.2, and 71.3 mg/ml for samples (MgB_2_-PVP)^foil^_0.25_, (MgB_2_-PVP)^foil^_3_, (MgB_2_-PVP)^tube^_0.25_, and (MgB_2_-PVP)^tube^_3_, respectively. These values are comparable, or they are one order of magnitude higher that the MIC and MBIC values mentioned in “Cytocompatibility of MgB_2_ powders”. They are also about three orders of magnitude larger than the toxicity limit of the powders. Interaction of MgB_2_ with water, culture media, or body fluids is complex and needs further investigations. Some information for Mg, MgO, Mg(OH)_2_ and MgB_2_ was reported in Refs.^26,27,36,39–41^. The involved processes develop in multistep sequences specific for each phase. Moreover, the reaction products, kinetic factors, and environmental conditions will ensure, or not, the background for antibacterial activity evolution with time. For example, the reaction of MgB_2_ with water leads to formation of Mg(OH)_2_, while boron in water forms boric acid. Due to low solubility in water, Mg(OH)_2_ can passivate the surface of MgB_2_ decreasing its efficiency. However in physiologically relevant solutions Mg(OH)_2_ was found to dissociate^32^. On the other hand, in the reaction between MgB_2_ and water, the H_2_-gas is released, and it can disrupt the passivating layer and can clean the surface of the MgB_2_ particle. The overlapping of the processes, some of them with possibly opposite contributions, can explain the non-linear dependence of the bioactivity with time observed in our experiments from “Antibacterial activity of MgB_2_-PVP composite coatings”, but one should also consider the decreasing amount of MgB_2_, which is consumed in the interaction with cells and environment. Therefore, the current discussion and provided numbers draw attention on the necessity to properly design the coatings considering each specific application.

The mechanisms by which our composite coatings develop the antimicrobial effect deserves further targeted studies. In general, for nanomaterials the mechanisms^11,42^ are related to their influence on production of reactive oxygen species (ROS) (and in some cases of *reactive nitrogen species* RNA) through a catalytic action described by Haber/Weiss- and Fenton- type reactions^13,43^, on electrostatic interaction that can impact the disruption/damage of the membrane integrity and potential so that metabolic functions of the cells are affected, and on the changes in the local environmental conditions e.g. through modification of aeration and pH. Depending on materials, cells, and environment there are specific features. For our composite coatings, PVP disolves in water, MgB_2_ is released and it becomes active interacting with the aqueous environment^44^ and the cells (Fig. 2IV). PVP is inert in respect to its antimicrobial activity^45^. The possibility of exfoliation of MgB_2_ with formation of nano sheet materials such as hydroxyl functionalized Mg-deficient MgB_2_^36^ or borophane (HB)^37^ may provide extra positive specific value in fighting the microbes: the 2D borophene-type materials are shown to have catalytic properties^46^ that can impact directly or indirectly also bioprocesses. Furthermore, another 2D material, namely the graphene oxide emerged as an effective antimicrobial material^47^ and, through similarities and extrapolation, one may expect that also the 2D nano boron-type materials derived from MgB_2_ will play an important role as antimicrobial materials. However, at present, there are no experiments performed in this direction. In summary, different ionic species are generated because of the MgB_2_ presence and they are at the origin of the developing reactions necessary for the antimicrobial effect (Fig. 2V).

The antimicrobial activity of MgB_2_ assessed in our work revealed good efficiency against selected bacteria, e.g. for planktonic growth (Fig. 5) one may observe a decrease for (MgB_2_-PVP)^foil^_3_ of 7log/24 h against *E. coli*, 8log/24 h against *P. aeruginosa*, 8log/24 h against *S. aureus*, and 5log/24 h against *E. faecium*. Literature present other effective composite antibacterial materials, such as AgBr/nPVP^45^ with a 5log/2 h against *E. coli*, and a lower concentration of the active substance (however, no citotoxicity data are available for comparison). In Ref.^48^ was reported a decrease of CFU/ml for nitric oxide (NO) of 8 log/24 h against *E. coli*/*A. baumannii*/*S. aureus*. In Ref.^49^ for copper (Cu) the decrease of CFU/ml was of 4log/24 h against *E. coli*/*S. aureus*. In Ref.^50^ was shown for silver (Ag) a decrease of CFU/ml of 5log/1.5 h against *E. coli*/*S. aureus*. It is important to note that a direct comparison between reported data is not entirely possible, as the assessment procedures are slightly different, the control samples do not start from the same level of cells population, and samples are different in shape and size and they target different applications.

## Conclusion

In vitro MTS and LDH cytotoxicity tests of the MgB_2_ activity on the HS27 dermal cell line indicated a toxicity limit in the range of 8.3–33.2 µg/ml depending on the powder type. Three powders were tested and the most active was LTS. According to literature^23^ this powder shows the highest amount of MgB_2_ phase, impurity-free particle surfaces, the largest fraction of the particle size in the nano-range and the highest rate of pH increase. The LTS powder was further used for fabrication of MgB_2_–PVP coatings on plastic elements from a commercial urinary catheter. The as-prepared self-replenishing and biodegradable MgB_2_–PVP composite coatings with two compositions were tested at 6, 24, and 48 h of incubation time against the bacterial growth of *Staphylococcus aureus* ATCC 25923, *Enterococcus faecium* DMS 13590, *Escherichia coli* ATCC 25922, *Pseudomonas aeruginosa* ATCC 27853. Strains were in the planktonic and biofilm growth states. The coatings were efficient both against planktonic microbes and microbial biofilms when applied on plain, flexible plastic foils. Results are promising and recommend MgB_2_ for designing efficient anti-infective solutions for various biomedical devices and systems.
